# *In vitro* genotoxicity assessment of French fries from mass catering companies: a preliminary study

**DOI:** 10.1093/mutage/geac021

**Published:** 2022-10-15

**Authors:** Julen Sanz-Serrano, Roncesvalles Garayoa, Ana Isabel Vitas, Adela López de Cerain, Amaya Azqueta

**Affiliations:** Department of Pharmacology and Toxicology, School of Pharmacy and Nutrition, Universidad de Navarra, Irunlarrea 1, 31008 Pamplona, Spain; Department of Nutrition, Food Science and Physiology, Universidad de Navarra, School of Pharmacy and Nutrition, Irunlarrea 1, 31008 Pamplona, Spain; IdiSNA, Navarra Institute for Health Research, Irunlarrea 3, 31008, Pamplona, Spain; Department of Microbiology and Parasitology, School of Medicine, Universidad de Navarra, Irunlarrea 1, 31008, Pamplona, Spain; Department of Pharmacology and Toxicology, School of Pharmacy and Nutrition, Universidad de Navarra, Irunlarrea 1, 31008 Pamplona, Spain; IdiSNA, Navarra Institute for Health Research, Irunlarrea 3, 31008, Pamplona, Spain; Department of Pharmacology and Toxicology, School of Pharmacy and Nutrition, Universidad de Navarra, Irunlarrea 1, 31008 Pamplona, Spain; IdiSNA, Navarra Institute for Health Research, Irunlarrea 3, 31008, Pamplona, Spain

**Keywords:** mutagenicity, genotoxicity, deep-fried, French fries, fried potatoes, micronucleus test

## Abstract

It is generally assumed that French fries are likely to have weak *in vitro* mutagenic activity, but most studies thereof have only assessed gene mutations. In this article, the genotoxicity of 10 extracts of French fries was assessed using the *in vitro* micronucleus test (following the principles of the OECD 487 guidelines). Each sample was obtained from a different mass catering company in Navarra (Spain). This assay, together with the Ames test, is recommended in the basic *in vitro* phase included in the European Food Safety Authority Opinion on Genotoxicity Testing Strategies Applicable to Food and Feed Safety Assessment. Eight of 10 samples from mass catering companies induced chromosomal aberrations in the *in vitro* micronucleus test. Moreover, French fries deep-fried in the laboratory for different periods of time (0, 3, 5, 10, 20, 30 min) were assessed using the *in vitro* micronucleus test. Genotoxicity was observed in all time periods from 3 min on. The biological relevance of these results must be further explored.

**Highlights**

- This is the first genotoxicity assessment of French fries using the *in vitro* MN test.
- Ten deep-fried French fry samples from mass-catering companies were assessed.
- Eight samples (8/10) were positive by the *in vitro* MN test.
- Genotoxicity of French fries fried for different time periods was detectable from 3 min onward.

## Introduction

Potatoes are a staple of most diets, and, among other culinary techniques, they are commonly deep-fried before their ingestion both in food businesses and at home. Unfortunately, exposing potatoes to oils at high temperatures produce compounds related to cancerous processes in animals [1], including lipid peroxides, acrolein, 5-hydroxymethylfurfural, oxidized antioxidants, or the widely studied animal carcinogen acrylamide. In fact, acrolein and acrylamide are already classified as ‘probably carcinogenic to humans’ (Group 2A) by the International Agency for Research on Cancer (IARC) [2,3]. In this regard, the European Food Safety Authority (EFSA) began monitoring acrylamide content in food and foodstuffs after it was detected in frequently consumed cooked foodstuffs [4,5]. The food industry then made some effort to limit human exposure to acrylamide, but levels in food commodities were not systematically decreased, and the EFSA published a risk assessment guideline for acrylamide in food [6]. As a consequence, the European Commission established reference levels and concrete mitigation measures for the reduction of the presence of acrylamide in foodstuffs. This helps food manufacturers and businesses to guarantee the safety of their products and, therefore, minimize the health effects derived from this compound. The measures include maintaining frying temperatures below 175 °C, as well as frequently removing remains and crumbs from the oil [7].

The mutagenic activity of deep-fried French fries obtained from local establishments [8,9] and cooked in the laboratory [10,11] has been assessed by the bacterial reverse mutation test (Ames test). These studies generally conclude that the extracts have weak or no *in vitro* mutagenic activity. However, only one study tested the genotoxicity in assays other than the Ames test, i.e., an *in vitro* comet assay, which had negative results [8].

The EFSA states that adequate evaluation of the genotoxic potential of chemical mixtures, such as in the case of French fries, must cover the three genetic endpoints, i.e., gene mutations, and structural and numerical chromosomal alterations [12,13]. In this regard, the use of the Ames test and the *in vitro* micronucleus (MN) test is recommended as a first step when testing for genotoxicity. These assays should follow the corresponding Organization for Economic Co-operation and Development (OECD) guidelines [12].

The present study aims to assess the genotoxicity of French fries for the first time using the MN test, thus completing the EFSA *in vitro* genotoxicity evaluation strategy for complex mixtures. To do so, 10 extracts of French fries obtained from different mass catering companies in Navarra (Spain) were assessed for genotoxicity using the *in vitro* MN test following the OECD 487 guideline [14]. In addition, samples cooked in the laboratory for different time periods were tested.

## Material and methods

### Chemicals and reagents

The chemicals and reagents used include cell sorting set-up beads for blue lasers (beads) (Invitrogen, OR, USA), Dulbecco’s phosphate buffered saline (Gibco, Paisley, UK), heat-inactivated fetal bovine serum (FBS) (Gibco, NY, USA), penicillin-streptomycin (Lonza, Cologne, Germany), RNAase (Invitrogen, CA, USA), RPMI 1640 medium ‘American Type Culture Collection modified’ (Gibco, NY, USA), S9 fraction from livers of Aroclor 1254-induced rats (Moltox, NC, USA), and Sytox dye (Invitrogen, OR, USA). The remaining chemicals were obtained from Sigma–Aldrich (Steinheim, Germany).

### Potato samples

Samples were kindly provided by mass catering companies from different sectors in Navarra (Spain) (Table 1) and stored at −40°C for up to 7 months. Then, the extraction procedure was conducted (see Extraction procedure).

**Table 1.** List of French fries’ samples obtained from a different mass catering company from Navarra (Spain)

Sample	Sector	Cooking temperature	Oil	Weight after fried (g)^a^
1	Sanitary	160°C	High oleic sunflower	122.3
2	Education	N/D	Sunflower	122.2
3	Education	180°C	High oleic sunflower	102.9
4	Social	160°C	High oleic sunflower	76.2
5	Social	160°C	High oleic sunflower	70.4
6	Social	180°C	Sunflower	98.1
7	Education	170–180°C	Sunflower	62.9
8	Social	180°C	Sunflower	104.2
9	Social	150°C	High oleic sunflower	96.6
10	Social	N/D	N/D	92.3

N/D, no data.

Right before extraction (thawed).

In addition, precooked frozen French fries were purchased from a local supermarket, and 200 g from each was deep-fried in high oleic sunflower oil for 3, 5, 10, 20, and 30 min at 175 °C. Non-fried precooked frozen French fries were also included in the assays (referred to as 0 min). Samples were then ground down and stored in a flask at −40°C until the extraction procedure was conducted. Raw potatoes were purchased from the same supermarket, and after being peeled and sliced, 200 g were subjected to extraction (referred to as raw).

### Extraction procedure

Frozen samples were thawed and homogenized two to three times v/w 95% methanol and maintained for 24 h in continuous agitation. The mixture was then centrifuged at 5,251 × g for 20 min. The supernatant was filtered through glass wool and stored at 4°C. The resulting sediment was suspended again in two to three times v/w 95% methanol and maintained in continuous agitation for additional 24 h. After being filtered through glass wool, both supernatants were then combined, and the solvent was evaporated. The viscous solid was then mixed with 4 ml of DMSO and stored at −40°C.

### Cell culture

Human lymphoblastoid TK6 cell-line (ATCC, VA, USA) was used to perform the MN test, as recommended by OECD guideline 487 [14]. The suspension culture (0.2–1 × 10^6^ cells/ml) was maintained in a supplemented RPMI 1640 medium (10% FBS, 100 U/ml penicillin and 100 µg/ml streptomycin), continuously agitated and kept at stable 37°C with 5% CO_2_ for up to 60 days. During the experimental period, cell doubling time was approximately 15 h.

### *In vitro* MN test

The recommendations in OECD guideline 487 [14] were followed to perform the *in vitro* MN assay.

Extracts and consecutive 1/2 dilutions derived from deep-fried samples obtained from mass catering companies were assessed for 4 h in the absence and presence of metabolic activation (S9−/S9+) and for 24 h in the absence of metabolic activation (S9−). Moreover, extracts obtained from raw potatoes and precooked frozen French fries deep-fried in the laboratory for 0 (non-fried), 3, 5, 10, 20, 30 min were assessed for 24 h (S9−) in two independent experiments.

#### Test procedure

The procedure was previously described [15]. S9 fraction (1%) and cofactors β-nicotinamide adenine dinucleotide phosphate (NADP) sodium salt hydrate (1.5 mg/ml) and dl-isocitric acid (2.7 mg/ml) were used as the external metabolic activation system. Cyclophosphamide (CP, 4 µg/ml) and colchicine (COL, 10 ng/ml) were used as positive controls for 4 h treatment in the presence of S9 and for 24 h treatment, respectively. DMSO (1%) was used as a negative (solvent) control. Baseline MN levels in the negative controls were monitored and did not vary throughout the experimental period.

Briefly, cells were treated in a 12-well plate with extract dilutions, positive control, or solvent for 24 h (i.e. until 1.5–2 cell cycles) in the absence of metabolic activation, or for 4 h in the absence or presence of metabolic activation, plus an additional 20 h of incubation. One replicate was treated per dilution of the extracts. Then, cells were centrifuged (141 × g, 8 min, 4 °C), and pellets were incubated in a cold EMA solution (0.025 mg/ml) for 20 min under 60 W direct light. After washing, cells were incubated in darkness at room temperature with lysis solution 1 (0.2 µM Sytox dye, 1 mg/ml RNAase, 0.584 mg/ml sodium chloride, 1 mg/ml trisodium citrate dihydrate, 0.3 µl/ml IGEPAL), and an additional 30 min after adding lysis solution 2 (0.2 μM Sytox dye, 1.5 μl/ml beads, 85.6 mg/ml sucrose, 15 mg/ml citric acid).

A fixed number of healthy nucleated cells (i.e. 20,000 ± 1000) were scored per condition in FACSCanto^TM^ II Six Colors (BD, NJ, USA). MN were determined using FlowJo^TM^ V10.2 software (BD, NJ, USA) following the MicroFlow Instructions Manual (Litron Laboratories, NY, USA).

#### Relative survival

Relative survival (RS) was used as a cytotoxicity indicator and calculated using the following formula:


$$\begin{array}{ll} RS= & (\frac{number\;of\;healthy\;cells}{number\;of\;beads}\;/\;\frac{\begin{array}{c} number\;of\;healthy\;cells\;\\ \;in\;negative\;control \end{array}}{\begin{array}{c} number\;of\;beads\\ \;in\;negative\;control \end{array}})\times 100 \end{array}$$

Each condition contained a fixed number of beads derived from Lysis solution 2. These beads were counted by the cytometer (*number of beads*) until reaching 20,000 healthy nuclei (*number of healthy cells*). Then, each condition was compared with the negative control and RS was shown as a percentage.

#### Criteria for a positive result

All of the following criteria must be fulfilled in any of the conditions to consider an extract to induce MN:

a) Statistically significant MN induction in at least one of the non-cytotoxic concentrations (i.e. RS > 40%) compared with the negative control value.
b) At least one of the non-cytotoxic concentrations induces a three-fold MN increase compared with the negative control value.
c) MN induction follows a statistically significant and concentration-related increase over the non-cytotoxic range tested

### Statistical analysis

The Statistics and Data Analysis (STATA) software v12.1 (TX, USA) was used to analyze data and trends with the chi-square test and simple linear regression, respectively. Statistical significance was set at *p* ≤ 0.05.

## Results

The *in vitro* genotoxicity of extracts from 10 French fry samples obtained from different mass catering companies and cooked under commercial conditions was assessed using the *in vitro* MN test (OECD 487) [14]. The specifications of each sample are described in Table 1. Each sample corresponds to a standard portion of French fries randomly collected from mass catering companies to avoid cooking biases; therefore, no cooking times are shown. Raw potatoes and French fries deep-fried in the laboratory at different times under controlled conditions were also assessed using the MN test after 24 h of treatment.

### French fry samples from mass catering companies

The genotoxicity of extracts from French fry samples obtained from mass catering companies was evaluated using the MN test after 4 h (S9−/S9+) and 24 h (S9−) in TK6 cells; results are shown in Table 2. For the 4-h treatment, extracts 3 (S9+) and 10 (S9−) showed a statistically significant threefold MN increase at non-cytotoxic concentrations compared with the solvent control and a statistically significant trend. After 24 h, all the extracts, except for samples 5, 9, and 10, induced a three-fold MN increase at non-cytotoxic concentrations and a statistically significant trend.

**Table 2.** Results of the MN test after TK6 exposure to serial dilutions of extracts derived from French fries’ samples fried in mass catering companies. The experiments were conducted for 4 h with and without metabolic activation (S9+/S9−) and 24 h without metabolic activation (S9−). MN have been analyzed by flow cytometry in 20,000 cells. MN per 10^3^ nucleated cells and relative survival rate (RS, in brackets) are shown for each condition.

4 h (S9−/S9+)										
Dil.	Extract 1		Extract 2		Extract 3		Extract 4		Extract 5	
	S9−	S9+	S9−	S9+	S9−	S9+	S9−	S9+	S9−	S9+
**0**	7.7 (100)	9.1 (100)	8.8 (100)	7.7 (100)	4.4 (100)	3.9 (100)	5.8 (100)	5.7 (100)	7.7 (100)	8.5 (100)
**1/8**	9.8* (111)	8.1 (104)	6.5 (114)	8.0 (104)	5.8 (93)	6.9*** (79)	7.8* (97)	8.5** (118)	7.8 (101)	6.6 (117)
**1/4**	6.5 (98)	5.6 (95)	6.4 (114)	8.6 (89)	6.0* (88)	6.1** (82)	5.7 (110)	7.4* (100)	5.6 (99)	6.2 (118)
**1/2**	8.6 (88)	11.8** (100)	6.9 (119)	8.3 (102)	4.8 (94)	6.6*** (84)	5.7 (109)	8.2** (83)	7.5 (98)	6.9 (90)
**1/1**	40.7*** (14)	10.4 (78)	8.2 (100)	17.6*** (91)	11.1*** (61)	12.7*** (61)	8.6** (104)	10.4*** (82)	7.5 (86)	27.9*** (61)
**Trend**	ns	ns	ns	*	ns	*	ns	ns	ns	ns

	Extract 6		Extract 7		Extract 8		Extract 9		Extract 10	
	S9−	S9+	S9−	S9+	S9−	S9+	S9−	S9+	S9−	S9+
**0**	5.1 (100)	4.4 (100)	9.5 (100)	8.1 (100)	7.4 (100)	11.6 (100)	5.7 (100)	7.7 (100)	5.1 (100)	7.1 (100)
**1/8**	10.3*** (94)	6.7** (105)	12.1* (84)	9.2 (105)	8.0 (88)	6.2 (70)	6.1 (59)	9.0 (67)	13.5*** (59)	9.8** (86)
**1/4**	8.1*** (101)	8.0*** (102)	6.7 (93)	9.0 (117)	10.0** (65)	6.1 (67)	7.8* (63)	8.8 (50)	9.4*** (57)	12.3*** (79)
**1/2**	7.6** (89)	4.9 (89)	13.2** (73)	9.1 (128)	9.5* (81)	12.2 (44)	8.1** (70)	10.7** (69)	13.2*** (57)	11.0*** (48)
**1/1**	8.3*** (89)	70.0*** (25)	24.0*** (42)	16.3*** (100)	10.4** (81)	9.7 (62)	13.6*** (55)	12.4*** (32)	20.2*** (46)	— (0)
**Trend**	ns	ns	*	*	ns	ns	**	*	*	ns

24 h (S9−)										
Dil.	Extract 1		Extract 2		Extract 3		Extract 4		Extract 5	
	MN	RS	MN	RS	MN	RS	MN	RS	MN	RS
**0**	5.0	(100)	4.3	(100)	3.9	(100)	3.9	(100)	3.7	(100)
**1/16**	4.5	(94)	6.1*	(120)	5.3**	(117)	6.2**	(92)	6.0**	(78)
**1/8**	7.2**	(92)	6.9**	(139)	4.8	(102)	4.9	(91)	5.1*	(134)
**1/4**	7.6**	(72)	9.6***	(116)	5.0	(128)	7.7***	(100)	5.0	(139)
**1/2**	19.1***	(52)	7.0***	(111)	7.0***	(101)	13.9***	(91)	6.7***	(122)
**1/1**	135.8^a^	(15)	19.1***	(77)	17.2***	(53)	29.4***	(52)	8.5***	(93)
**Trend**	*		*		**		***		*	

	Extract 6		Extract 7		Extract 8		Extract 9		Extract 10	
	MN	RS	MN	RS	MN	RS	MN	RS	MN	RS
**0**	3.7	(100)	3.9	(100)	3.9	(100)	5.8	(100)	5.8	(100)
**1/16**	6.4***	(121)	4.9	(110)	2.6	(102)	9.4***	(104)	6.6	(105)
**1/8**	8.4***	(114)	6.5**	(103)	5.8**	(81)	6.1	(79)	7.9*	(88)
**1/4**	11.2***	(110)	5.3*	(98)	9.0***	(85)	14.3***	(79)	13.2***	(84)
**1/2**	14.5***	(95)	9.3***	(90)	5.9**	(79)	15.9***	(53)	15.6***	(55)
**1/1**	35.3***	(58)	21.6***	(58)	14.4***	(46)	51.5***	(30)	49.3***	(27)
**Trend**	***		**		*		ns		**	

Data not included in the statistical analysis; less than 20,000 cells were analyzed due to cytotoxicity.

Bold numbers indicate threefold increase compared to the negative control value. Positive controls: 4-h treatment with S9, CP = 62.4–353.5 MN/10^3^ cells. 24-h treatment, COL = 104.4–407.6 MN/10^3^ cells.

Statistical significance compared to negative control (i.e. 0) was set as: ns *P* > 0.05; *P ≤ 0.05; **P ≤ 0.01; ***P ≤ 0.001.

### Potato samples cooked in the laboratory

Extracts from raw potatoes, and French fries deep-fried for 0, 2, 5, 10, 20, and 30 min, were prepared and evaluated for genotoxicity in TK6 treated for 24 h without metabolic activation (Table 3). Regarding edibility, French fries were crispy (3–5 min) or dry/overcooked (10–30 min). All the extracts from 3- to 30-min deep-fried French fries showed a statistically significant three-fold MN increase in non-cytotoxic concentrations, and a concentration-response trend, in both independent experiments. This trend was not statistically significant in some cases. French fries that were not deep-fried in the laboratory (0 min) exerted a three-fold MN increase in the highest non-cytotoxic concentration and a statistically significant dose–response trend, but these results were not confirmed in the second experiment. Raw potatoes did not induce MN in any of the experiments.

**Table 3.** MN test after TK6 exposure to serial dilutions of extracts derived from raw potatoes or French fries deep-fried in the laboratory for 0, 2, 5, 10, 20, and 30 min. Treatment time of independent experiments was 24 h without metabolic activation (S9−). MN have been analysed by flow cytometry in 20,000 cells. MN per 10^3^ nucleated cells and relative survival rate (RS, in brackets) are shown for each condition.

Dil.	Raw	0 min	3 min	5 min	10 min	20 min	30 min
**Experiment 1**							
**0**	2.1 (100)	2.1 (100)	3.3 (100)	3.3 (100)	3.3 (100)	3.3 (100)	3.3 (100)
**1/8**	2.5 (93)	2.4 (112)	6.1*** (104)	3.1 (165)	3.9 (120)	3.6 (120)	2.5 (130)
**1/4**	3.0 (83)	1.7 (103)	26.0*** (74)	3.7 (132)	6.4*** (127)	4.2 (118)	2.8 (150)
**1/2**	2.8 (72)	3.5* (93)	128.4*** (37)	10.8*** (78)	16.9*** (59)	10.7*** (51)	4.9*** (85)
**1/1**	4.8*** (52)	9.0*** (60)	**699.1** ^a^ (12)	42.5*** (56)	178.0*** (29)	**31.8** ^a^ (13)	12.5*** (70)
**Trend**	*	*	ns	*	ns	ns	*
**Experiment 2**							
**0**	2.0 (100)	2.0 (100)	1.0 (100)	1.0 (100)	1.0 (100)	1.0 (100)	1.0 (100)
**1/8**	2.9 (75)	1.2 (110)	4.9*** (79)	2.2** (114)	2.0* (118)	3.6*** (95)	1.3 (119)
**1/4**	2.4 (101)	1.5 (101)	28.0*** (40)	3.3*** (105)	4.6*** (87)	2.7*** (94)	2.0* (94)
**1/2**	4.2*** (78)	3.2* (81)	207.0*** (23)	8.8*** (82)	12.4*** (76)	9.0*** (50)	4.0*** (71)
**1/1**	5.7*** (41)	5.8*** (61)	268.9^a^ (9)	41.0*** (43)	161.3*** (28)	28.8*** (10)	7.5*** (47)
**Trend**	**	*	ns	*	*	ns	***

Data not included in the statistical analysis; less than 20,000 cells were analyzed due to cytotoxicity.

Bold numbers indicate threefold increase compared with the negative control value. Positive control: 24 h: COL (S9−) 31.8–58.8 MN/10^3^ cells.

Statistical significance compared with negative control (i.e. 0) was set as: ns *P* > 0.05; *P ≤ 0.05; **P ≤ 0.01; ***P ≤ 0.001.

## Discussion

Due to the scarcity of information related to the genotoxicity of French fries, the current article evaluated deep-fried French fry extracts using the *in vitro* MN test following the recommendations of OECD guideline 487 [14]. This guideline regards as optional the present flow-cytometry-based method, and it has been shown to be at least as equally reliable as the standard microscopy method [16].

Regarding previous evidence, it seems that the genotoxicity of French fry extracts induce weak or no mutagenicity in the Ames test and no strand breaks in the comet assay [8–11]. The current article complements the available evidence with recommendations from EFSA’s genotoxicity testing strategies by performing the *in vitro* MN test. To our knowledge, this is the first time that the *in vitro* MN test is used to evaluate the genotoxicity of deep-fried potato extracts.

There is no consensus on the best extraction method for yielding the mutagenic and genotoxic compounds present in French fries. The alkaline extract is reported to be capable of inducing mutagenic activity in the Ames test, while the neutral and acidic fractions are not [9,10], nor are the aqueous and organic extracts [8]. Therefore, in this study, a simple but effective method was used for the preparation of the extracts based on the hydrophilic weak-base-nature of the genotoxic compounds characterized in French fries. Commonly, these polar substances, such as acrylamide, easily dissolve in water or other polar solvents, e.g., methanol [17].

In addition, each sample obtained from mass catering companies was used in its entirety in order to maximize the sensitivity of the assay (Table 1). All samples weighed 100 ± 25 g except for samples 5 and 7, which weighed less. In any case, different concentrations of the extracts were prepared and checked to assess a dose-response effect and toxicity. French fry samples were fried at temperatures in which acrylamide and other genotoxic compounds are commonly formed (i.e. >120°C) [1,18]. Although cooking duration data were not collected, it is assumed that all samples were adequately prepared for consumption.

Eight of 10 French fry samples obtained from mass catering companies were found positive in the MN test. These positive outcomes were then subjected to cytotoxicity interpretation since MN could have been induced based on mechanisms not relevant to genotoxicity [19]. In this regard, OECD guideline 487 states that care should be taken in interpreting positive results only found in the higher end of 55 ± 5% of cytotoxicity [14]. When using the flow-cytometry-based method, RS has been found to be the most reliable cytotoxicity parameter in defining the selection of top concentration compared with relative cell count and relative population doubling, as recommended by OECD guideline 487 [16]. Once the cytotoxic results (RS < 40%) were discarded, eight extracts were considered positive in the MN test, i.e., samples 1, 2, 3, 4, 6, 7, 8, and 10. All of them showed a positive response after 24 h of treatment except for sample 10, which was positive after 3 h of treatment in the absence of metabolic activation. Sample 3 was also positive after 3 h in the presence of metabolic activation. As previously mentioned, sample 5 and 7 weighed less than the rest; while sample 7 was clearly positive, sample 5 induced mild cytotoxicity for all of the conditions tested. This may imply that a more concentrated extract exerts genotoxicity.

Extracts from raw potatoes and French fries deep-fried for 0, 2, 5, 10, 20, and 30 min were also assessed for 24 h (S9−) (Table 3). Weight, whether raw potatoes or French fries prior to the frying process, was standardized to 200 g. Raw potatoes did not induce MN and the non-fried frozen French fry sample (0 min) exerted disparate results between the two experiments, one being clearly positive and the other one negative. Nonetheless, MN induction in the second experiment (i.e. the one showing negative results) was almost three-times the value in the negative control. This outcome can be explained because these samples were subjected to heat treatment before they are frozen and commercialized. In any case, further deep-frying enhanced the genotoxicity in all the samples; all extracts from French fries deep-fried for 3–30 min showed a statistically significant three-fold MN increase in non-cytotoxic concentrations. A high level of MN was observed at the highest non-cytotoxic concentrations in some of the conditions tested. This can explain why the dose–response trend was not significant in some cases (Table 3). It is worth mentioning that samples were crispy after 3–5 min and dry/overcooked (i.e. inedible) after 10–30 min.

The results obtained in this study and those available in the literature on the genotoxicity of French fry extracts may be partially related to the presence of acrylamide, whose genotoxicity has been extensively studied [6]. Acrylamide is a weak mutagen in bacterial systems, but an effective clastogen and aneugen in the absence of metabolic activation [6,20]. The general population is mainly exposed to acrylamide via French fries, chips, coffee, or bread, which is formed during frying or baking processes [21]. In humans, acrylamide is known to be metabolized by cytochrome P450 2E1 (CYP2E1) to glycidamide, which is responsible for its *in vivo* genotoxicity and probable carcinogenicity via the DNA adduct mechanism [6,22]. In addition to acrylamide, other potential compounds could contribute to the genotoxicity observed in fries. The caramelization products in starch, potatoes’ major component after water [10], could have clastogenic activity [23]; caramelized sugars induced a high frequency of chromosome breaks and exchanges in cultured CHO after 3 h of exposure while non-caramelized ones did not. Alkanes, ketones, alcohols, furans, aldehydes, and other breakdown products are formed by lipid peroxidation, which can also interact with proteins and nucleic acids [1,24]. Among them, malonaldehyde, 4-hydroxynonenal (HNE), and acrolein are the best studied. Malonaldehyde is the major product of lipid peroxidation, and it has mutagenic potential in Ames testing, especially in base pair strains [24], but it has not been studied for chromosomal aberrations. HNE is also especially relevant as it is the main aldehydic peroxidation product and a strong alkylating agent [24]. The clastogenic effect of HNE has been previously reported *in vitro* [25–27], although a more recent study reported no micronucleus induction in an interlaboratory approach [28]. Moreover, HNE lacked *in vivo* micronucleus induction in mice [29]. The available literature on the *in vitro* genotoxicity of acrolein is inconsistent and controversial, although it is well known that it forms adducts with all DNA bases *in vitro* [1,24], and it increased the frequency of micronucleus in rats in a recent study [30].

The use of a non-standardized extraction method is one of the most significant limitations when assessing non-characterized complex mixtures like French fries. Unfortunately, there is still not enough evidence to establish the optimal extraction method for all genotoxic compounds present in French fries. The method presented in this article relies on a commonly used solvent to prioritize the yield of a wide range of compounds, rather than focusing on a certain known group of chemicals. Thus, it lays the foundation for further development.

Evaluating genotoxicity using only one assay, i.e., the MN test, can also be regarded as a limitation to this study. However, as previously mentioned, French fry extracts induce weak or no mutagenicity in the Ames test, although a complete Ames test has not been performed, and do not induce strand breaks in the comet assay [8–11]. Therefore, this preliminary study aimed to complete the existing information by performing the MN test, an assay that has never been used before, although it is included in the EFSA genotoxicity strategy.

Furthermore, testing extracts implies exposure risks; cells *in vitro* might be exposed to greater concentrations than *in vivo*, leading to a greater probability of misleading effects and positive results that are not relevant to humans. In this regard, it is essential to note that genotoxicity is still commonly considered a categorical variable rather than a continuous variable in regulatory assessment, thus emphasizing the genotoxic/non-genotoxic duality, rather than establishing a concentration threshold. However, the results observed in this preliminary work call for more studies to address these limitations and to further explore the biological relevance of these results. Although the EFSA strategy typically recommends an *in vivo* follow-up after a positive result in *in vitro* studies, this would hardly be feasible when testing food mixtures. The elevated number of animals needed for all samples from mass catering companies would imply significant ethical problems for testing based on the current trend to reduce the number of animals involved in experimentation. Therefore, other methods that better resemble humans’ physiological conditions should be considered, such as using other sources of metabolic activation or testing samples subject to *in vitro* digestion and/or fermentation processes.

## Conclusion

The available information on the mutagenicity of French fries, scarce as it is, concludes that French fries possess weak or no *in vitro* mutagenicity activity and do not induce strand breaks. Following OECD guideline 487, this study evaluated deep-fried French fry extracts using the *in vitro* MN test to detect chromosome aberrations for the first time. Therefore, it complements the available evidence with recommendations from the EFSA’s genotoxicity testing strategies. Eight out of ten French fry samples obtained from mass catering companies induced chromosomal aberrations using the *in vitro* MN test. Furthermore, deep-fried French fries were genotoxic in all frying time periods from 3 min onward. In view of this outcome, more *in vitro* studies like the ones discussed in this article are needed to further explore the human relevance of these results.
